# Langzeitergebnis nach akutem dialysepflichtigem Nierenversagen auf einer internistischen Intensivstation

**DOI:** 10.1007/s00063-020-00719-7

**Published:** 2020-08-21

**Authors:** L. Mizera, M. M. Dürr, D. Rath, F. Artunc, M. Gawaz, R. Riessen

**Affiliations:** 1 Medizinische Klinik III – Kardiologie und Angiologie, Universitätsklinikum Tübingen, Otfried-Müller-Str. 10, 72076 Tübingen, Deutschland; 2 Interdisziplinäre Intensivstation, Medizinisches Universitätsklinikum Tübingen, Tübingen, Deutschland; 3 Medizinische Klinik IV – Diabetologie, Endokrinologie, Nephrologie, Universitätsklinikum Tübingen, Tübingen, Deutschland

**Keywords:** Akutes Nierenversagen, Dialyse, Nierenersatztherapie, Nierenfunktion, Überleben, Acute kidney injury, Dialysis, Renal replacement therapy, Kidney function, Survival

## Abstract

**Hintergrund:**

Das akute dialysepflichtige Nierenversagen („dialysis-requiring acute kidney injury“ [AKI‑D]) ist eine häufige und schwerwiegende Komplikation bei intensivmedizinisch behandelten Patienten.

**Fragestellung:**

Im Rahmen dieser Studie sollte untersucht werden, welchen Einfluss ein AKI‑D auf die Sterblichkeit von Intensivpatienten besitzt, bei welchem Anteil der überlebenden Patienten auch bei Entlassung noch ein Nierenersatzverfahren benötigt wird und wie sich dies auf die Langzeitmortalität und die längerfristige Notwendigkeit einer Dialysetherapie auswirkt.

**Material und Methoden:**

Auswertung von 118 Patientenfällen mit AKI‑D zwischen November 2016 und Dezember 2017 auf einer internistischen Intensivstation am Universitätsklinikum Tübingen. Die Dialysefreiheit zum Entlasszeitpunkt und die 1‑Jahres-Mortalität wurden als primäre Endpunkte definiert. Den sekundären Endpunkt stellte die Dialysepflichtigkeit nach 18 Monaten dar.

**Ergebnisse:**

Die Krankenausmortalität der Patienten mit AKI‑D betrug 45,8 % (54/118). Von den 64 überlebenden Patienten mit AKI‑D waren 41 (64,1 %) zum Zeitpunkt der Entlassung nicht mehr auf ein Nierenersatzverfahren angewiesen. Im Vergleich dazu war die 1‑Jahres-Mortalität bei den 23 (35,9 %) Patienten, bei denen zur Krankenhausentlassung noch eine Dialysepflicht bestand, signifikant höher (24,4 % vs. 60,9 %, *p* = 0,004). Eine Dialysepflichtigkeit 18 Monate nach Krankenhausentlassung bestand bei 7 Patienten (10,9 %). Zu diesem Zeitpunkt waren überwiegend noch Patienten von einer Dialyse abhängig, bei denen bereits zum Entlasszeitpunkt ein Nierenersatzverfahren notwendig war (71,4 % vs. 7,1 %, *p* = 0,001).

**Schlussfolgerungen:**

Schwere AKI-Episoden, bei denen ein Nierenersatzverfahren auf einer Intensivstation notwendig wird, sind auch 1 Jahr bzw. 18 Monate nach Entlassung mit einer erhöhten Mortalität bzw. einer anhaltenden Dialysepflichtigkeit assoziiert.

## Abstract (en)

**Background:**

Acute kidney injury (AKI) is a common and serious complication of acute life-threatening diseases.

**Objectives:**

The aim of this study was to investigate the effect of acute renal failure on mortality in intensive care patients, the need for renal replacement therapy at discharge, and the effect on long-term mortality.

**Material and methods:**

Evaluation of 118 patient cases with dialysis-dependent acute renal failure between November 2016 and December 2017 admitted to a medical intensive care unit (ICU) at the University Hospital Tübingen, Germany. Dialysis at discharge and 1‑year mortality were defined as the primary endpoints. The secondary endpoint was need for continuous renal replacement after 18 months.

**Results:**

In 118 patients, renal replacement modality by means of hemodialysis became necessary. A mortality rate of 45.8% (54/118) was found in patients requiring dialysis. Of the 64 surviving dialysis-dependent patients, 35.9% were still dependent on renal replacement therapy at the time of discharge. The 1‑year mortality rate was significantly higher in patients that still required dialysis at the time of discharge (*p* = 0.004). At 18-month follow-up, seven patients (10.9%) were still on renal replacement therapy. At this time, dialysis was significantly more frequent in patients with dialysis at the time of discharge than in dialysis-free patients (7.1% vs. 71.4%, *p* = 0.001).

**Conclusion:**

Severe episodes of AKI requiring renal replacement therapy in the setting of an ICU are associated with increased mortality 1 year after discharge and an increased requirement for renal replacement 18 months after discharge.

## Einleitung

Ein akutes Nierenversagen („acute kidney injury“ [AKI]) tritt häufig im Rahmen einer schweren, intensivmedizinisch behandelten Erkrankung auf und ist mit einer erhöhten Mortalität assoziiert [3, 9, 17]. Patienten mit AKI, bei denen ein Nierenersatzverfahren („dialysis-requiring acute kidney injury“ [AKI-D]) notwendig wird, weisen dabei die höchste Mortalität auf [16]. Auch bei erholter Nierenfunktion zum Entlasszeitpunkt besteht ein erhöhtes Risiko, im Verlauf erneut ein dialysepflichtiges Nierenversagen zu entwickeln [16]. Vorteile verschiedener Dialysemodalitäten (intermittierend vs. kontinuierlich, diffusiv vs. konvektiver Transport, Dialysedosis) auf die Prognose oder die Nierenfunktion konnten bisher nicht gezeigt werden [15]. Auch nach einer AKI-Episode ohne Dialysepflichtigkeit ist das Mortalitätsrisiko 90 Tage nach Entlassung noch erhöht [10]. Bisher liegen allerdings wenige Daten über die Langzeitprognose kritisch kranker Patienten mit schweren Fällen eines AKI, bei denen ein Nierenersatzverfahren notwendig wurde, vor [1, 13, 16]. Diese Studie untersuchte an einem Kollektiv von internistischen Intensivpatienten, inwiefern sich die Nierenfunktion nach einem akuten dialysepflichtigen Nierenversagen 1 Jahr nach Entlassung von einer internistischen Intensivstation bzw. aus dem Krankenhaus wieder erholen kann oder ob noch dauerhaft ein Nierenersatzverfahren benötigt wird. Weiterhin galt es, die Langzeitprognose abzuschätzen und mögliche Risikofaktoren für eine bestehende Dialysepflichtigkeit nach einem AKI zu ermitteln.

## Methoden

**Figure 1:**
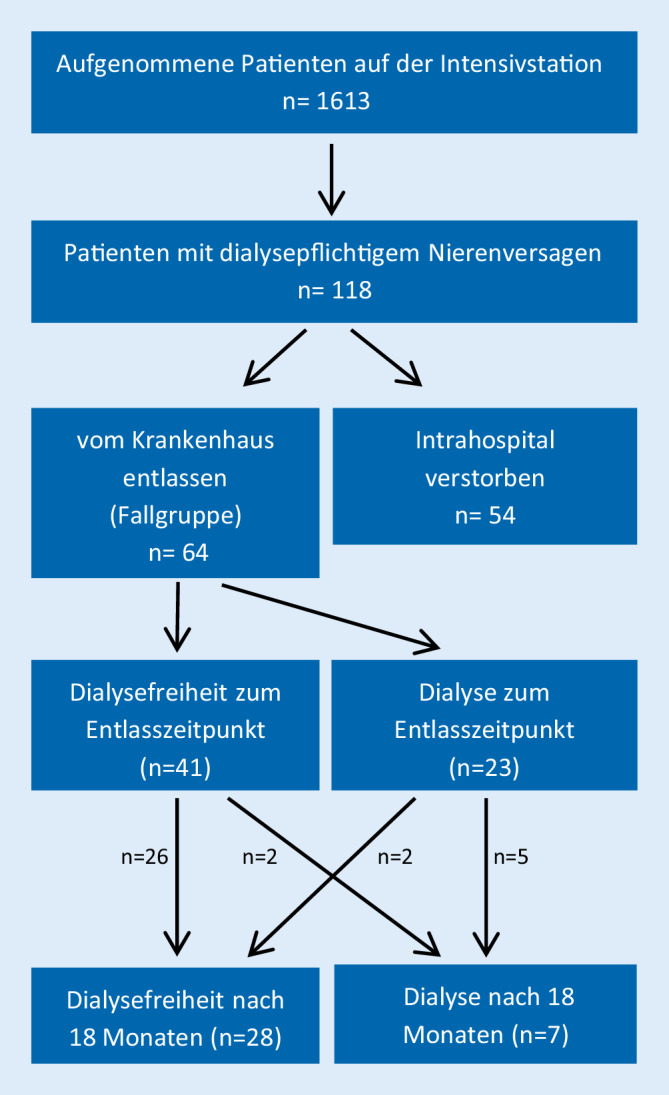

Das Studienprotokoll wurde von der Ethikkommission genehmigt. 118 konsekutive Patienten mit dialysepflichtigem akutem Nierenversagen wurden in diese Studie eingeschlossen. Alle Patienten wurden zwischen November 2016 und Dezember 2017 auf der internistischen Intensivstation des Universitätsklinikums Tübingen aufgenommen. Die Dialysebehandlung erfolgte als „slow extended dialysis“ (SLED) mittels des Genius-Systems und/oder als eine kontinuierlich venovenöse Hämodialyse (CVVHD) mittels CiCa (beide Fresenius Medical Care, Bad Homburg vor der Höhe, Deutschland) nach klinischem Bedarf. Die Patienten mit dialysepflichtigem Nierenversagen, welche nicht während des Aufenthalts verstorben sind (Fallgruppe), wurden mithilfe der elektronischen Krankenakten hinsichtlich vorbestehender Niereninsuffizienz, weiterer Vorerkrankungen, des akuten Auslösers sowie eventuell begleitenden Organversagens und Laborparametern charakterisiert (Abb. 1). Ein besonderes Augenmerk lag dabei auf der Erholung der Nierenfunktion im Laufe des Aufenthalts, gemessen an einer Dialysefreiheit zum Zeitpunkt der Krankenhausentlassung sowie 18 Monate später. Zur Evaluation des Langzeitverlaufs wurde ein Fragebogen versendet oder die Patienten telefonisch kontaktiert. Von allen 118 Patienten konnten die Daten erhoben werden. Die Dialysefreiheit zum Entlasszeitpunkt und die 1‑Jahres-Mortalität stellten die primären Endpunkte dar. Die Dialysepflichtigkeit nach 18 Monaten stellte den sekundären Endpunkt dar. Weiterführend wurden die zwei Gruppen mit entweder Dialysefreiheit oder fortgesetzter Dialyse zum Entlasszeitpunkt näher untersucht. Zur statistischen Auswertung wurde SPSS 25.0 (SPSS; IBM, Armonk, NY, USA) genutzt. Kontinuierliche Variablen wurden als Mittelwert ± Standardabweichung dargestellt und mit dem T‑Test verglichen. Wir analysierten qualitative Daten unter Verwendung des Chi-Quadrat-Tests. Unabhängige Prädiktoren für die Mortalität wurden mithilfe der Cox-Regressionsanalyse berechnet. Zusätzlich erstellten wir eine Kaplan-Meier-Kurve, um Assoziationen zwischen der 1‑Jahres-Mortalität und der Dialysefreiheit zum Entlasszeitpunkt darzustellen.

## Ergebnisse

Insgesamt wurden in dem Beobachtungszeitraum November 2016 und Dezember 2017 1613 Patientenfälle auf der internistischen Intensivstation behandelt. Von diesen hatten 118 Patienten (7,3 %) ein AKI‑D. Von den 118 Patienten mit AKI‑D starben 45,8 % (*n* = 54) während ihres Aufenthalts im Krankenhaus. In der weiter analysierten Fallgruppe der überlebenden Intensivpatienten mit AKI‑D wurden 64 Patienten mit einem mittleren Alter von 65,3 Jahren eingeschlossen, bestehend aus 39 Männern und 25 Frauen (Tab. 1). Der primäre Endpunkt Dialysefreiheit zum Entlasszeitpunkt wurde von 41 (64,1 %) der 64 Patienten erreicht. 23 (35,9 %) Patienten waren auch zum Zeitpunkt der Entlassung noch auf ein Nierenersatzverfahren angewiesen.

**Table 1:** 

Charakteristika	Alle überlebenden Patienten mit AKI‑D (*n* = 64)	Dialysefreiheit zum Entlasszeitpunkt (*n* = 41)	Dialyse zum Entlasszeitpunkt (*n* = 23)	*p*-Wert
Alter (Jahre, MW ± SA)	65,3 ± 15,3	64,8 (±15,6)	66,2 (±15,2)	0,726
Verweildauer im Klinikum (Tage, MW ± SA)	36,8 (±25,7)	38,5 (±28,3)	33,9 (±20,5)	0,494
Verweildauer auf Intensivstation (Tage, MW ± SA)	11,3 (±13,3)	12,9 (±15,2)	8,3 (±8,3)	0,179
SAPS-Score	54,0 (±18,3)	57,9 (±17,2)	45,9 (±18,2)	*0,024*
*Vorerkrankungen, n (%)*				
Chronische Niereninsuffizienz	33 (51,6)	19 (46,3)	14 (60,9)	0,196
Myokardinfarkt	10 (15,6)	7 (17,1)	3 (13,0)	0,483
Herzinsuffizienz	13 (20,3)	7 (17,1)	6 (26,1)	0,292
Schlaganfall	6 (9,4)	6 (14,6)	0 (0,0)	0,060
Periphere arterielle Verschlusskrankheit	7 (10,9)	3 (7,3)	4 (17,4)	0,203
Malignom	22 (34,4)	14 (34,1)	8 (34,8)	0,585
Diabetes mellitus	19 (29,7)	12 (29,3)	7 (30,4)	0,570
Arterielle Hypertonie	39 (60,9)	23 (56,1)	16 (69,6)	0,215
Leberzirrhose	14 (21,5)	8 (19,5)	6 (26,1)	0,542
*Laborchemische Parameter (MW ± SA)*				
Kreatinin bei Aufnahme (mg/dl)	3,8 (±3,0)	3,45 (±2,32)	4,32 (±3,95)	0,267
Maximales Kreatinin (mg/dl)	5,4 (±2,6)	4,81 (±2,08)	6,52 (±3,17)	*0,011*
Harnstoff (mg/dl)	201 (±60,6)	205,4 (±54,5)	193,1 (±91,5)	0,500
*Anzahl der Organversagen, (MW ± SA)*	1,55 (±1,2)	1,8 (±1,1)	1,1 (±1,0)	*0,017*
*Ursächliche Faktoren für die AKI, n (%)*				
Sepsis, septischer Schock	21 (32,8)	18 (43,9)	3 (13,0)	*0,010*
Kardiogener Schock	9 (14,1)	5 (12,2)	4 (17,4)	0,412
Hypervolämie	6 (9,4)	5 (12,2)	1 (4,3)	0,290
Hepatorenales Syndrom	10 (15,6)	5 (12,2)	5 (21,7)	0,254
Hämorrhagischer Schock	5 (7,8)	2 (4,9)	3 (13,0)	0,242
Hypovolämie	4 (6,3)	2 (4,9)	2 (8,7)	0,455
Andere	9 (14,1)	4 (9,8)	5 (21,7)	0,171
*Dialyseverfahren, n (%)*				
Intermittierende Hämodialyse	48 (75,0)	28 (68,3)	20 (87,0)	
Citrat-CVVHD + intermittierende Hämodialyse	15 (23,4)	13 (31,7)	2 (8,7)	
Citrat-CVVHD	1 (1,6)	0 (0)	1 (4,3)	

Daten: Anzahl *n* (%)

*MW* Mittelwert, *SA* Standardabweichung, *SAPS*-Score Simplified Acute Physiology Score, *AKI* acute kidney injury, *CVVHD* continuous venovenous hemodialysis

Eine vorbestehende chronische Niereninsuffizienz war bei 33 (51,6 %) der 64 überlebenden Patienten dokumentiert. Bezüglich weiterer Komorbiditäten war bei 39 (60,9 %) Patienten ein Bluthochdruck bekannt. Ein Malignom war bei 22 (34,4 %) Patienten vorbeschrieben. Bei 10 (15,6 %) Patienten fand sich ein Myokardinfarkt und bei 13 (20,3 %) Patienten eine Herzinsuffizienz in der Vorgeschichte.

Die häufigsten ursächlichen Faktoren für das akute Nierenversagen stellten der septische Schock (32,8 %), das hepatorenale Syndrom (15,6 %) und der kardiogene Schock (14,1 %) dar (Tab. 2). Neben der Nierenfunktion waren in der Fallgruppe durchschnittlich 1,6 weitere Organversagen betroffen. Bei 50,0 % der Patienten lag zusätzlich eine Beeinträchtigung des Herz-Kreislauf-Systems vor. Ein respiratorisches Versagen bestand bei 25 (39,1 %) Patienten. Von einem Leberversagen waren 19 Patienten (29,7 %) betroffen. 63 Patienten (98,4 %) wurden während ihres Aufenthalts auf der medizinischen Intensivstation mit einem diskontinuierlichen Genius-System dialysiert. Hiervon erhielten 15 Patienten (23,4 %) im Vorfeld eine CVVHD mit regionaler Citratantikoagulation. Diese kam bei Patienten mit erhöhter Blutungsneigung und gestörter Hämostase entsprechend den Empfehlungen der Sektion Niere der DGIIN, ÖGIAIN und DIVI zum Einsatz [14]. Ein Patient (1,6 %) erhielt ausschließlich eine CVVHD zur Nierenersatztherapie.

**Table 2:** 

Organversagen	Alle überlebenden Patienten mit AKI‑D, *n* = 64 (%)	Dialysefreiheit zum Entlasszeitpunkt, *n* = 41 (%)	Dialyse zum Entlasszeitpunkt, *n* = 23 (%)	*p*-Wert
Herz/Kreislauf	32 (50,0)	23 (56,1)	9 (39,1)	0,149
Respiratorisch	25 (39,1)	19 (46,3)	6 (26,1)	0,091
Leber	19 (29,7)	13 (31,7)	6 (26,1)	0,430
Hämatologisch	23 (35,9)	19 (46,3)	4 (17,4)	*0,019*

Daten: Anzahl *n* (%)

**Figure 2:**
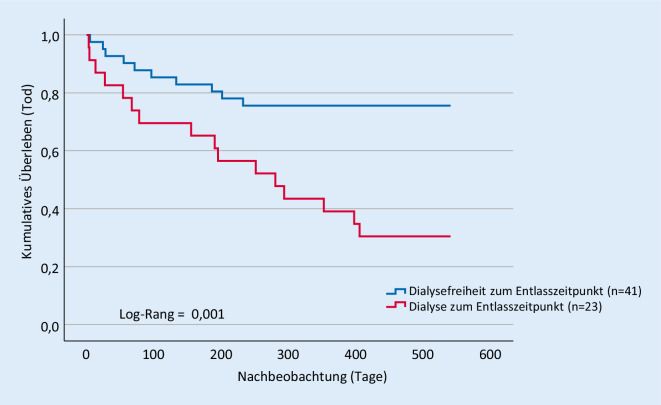

Innerhalb von 30 Tagen nach Krankenhausentlassung starben 7 (10,9 %) Patienten. Nach einem Jahr waren insgesamt 24 (37,5 %) der 64 vom Krankenhaus entlassenen Patienten mit AKI‑D verstorben. Die Kaplan-Meier-Kurve (Abb. 2) zeigt den Zusammenhang zwischen Überlebenswahrscheinlichkeit und der Notwendigkeit einer Dialyse zum Entlasszeitpunkt. Die Patienten ohne Dialyse zum Entlasszeitpunkt hatten eine signifikant höhere Überlebenswahrscheinlichkeit als die Patienten mit Dialyse (24,4 % vs. 60,9 %, *p* = 0,004). Insgesamt betrug die 1‑Jahres-Mortalität von den initial 118 Patienten mit AKI‑D auf unserer internistischen Intensivstation 66,1 %.

Anzahl der Ereignisse sowie Inzidenzrate pro 100 Patientenjahre sind in Tab. 3 dargestellt.

**Table 3:** 

Ereignis (*n*)	Dialysefreiheit zum Entlasszeitpunkt (ja vs. nein)	IR/100 PJ (ja vs. nein)	*p*-Wert
Dialysepflichtigkeit nach 18 Monaten (*n* = 7)	2/5	7,1/71,4	*0,001*
Mortalität nach 1 Jahr (*n* = 24)	10/14	24,4/60,9	*0,004*

*IR* Inzidenzrate, *PJ* Patientenjahre

Der SAPS-Score (54,0 ± 18,3 Punkte vs. 45,9 ± 18,2 Punkte, *p* = 0,024), die Anzahl der Organversagen (1,8 ± 1,1 vs. 1,1 ± 1,0, *p* = 0,017) und der septische Schock (18 vs. 3, *p* = 0,010) als auslösender Faktor für das akute Nierenversagen waren bei den zum Entlasszeitpunkt dialysefreien Patienten signifikant höher bzw. häufiger im Vergleich zu den Patienten, die zum Entlasszeitpunkt weiter dialysiert wurden. In dieser Gruppe war dagegen der Anteil der Patienten, die bereits eine vorbestehende chronische Niereninsuffizienz aufwiesen, mit 60,9 % (14/23) höher als in der dialysefreien Gruppe (46,3 %, 19/41). Allerdings war dieser Unterschied bei der geringen Fallzahl statistisch nicht signifikant (*p* = 0,196).

Bei 21,7 % (*n* = 5/23) der zum Entlasszeitpunkt dialysepflichtigen Patienten erholte sich die Nierenfunktion in den Folgemonaten, 78,2 % (*n* = 18/23) der Patienten blieben dauerhaft dialysepflichtig. Im weiteren Verlauf war bei 9,4 % (*n* = 6/41) der bei Entlassung dialysefreien Patienten nach initialer Dialysefreiheit erneut ein dialysepflichtiges Nierenversagen zu verzeichnen. Insgesamt 10,9 % (*n* = 7/64) der entlassenen Patienten erreichten lebend den sekundären Endpunkt Dialysepflichtigkeit nach 18 Monaten. Von diesen 7 Patienten gehörten 5 Patienten zur Gruppe der bei Entlassung dialysepflichten Patienten und 2 Patienten zu den zum Entlasszeitpunkt dialysefreien Patienten.

In der Cox-Regressionsanalyse war die Dialysefreiheit zum Entlasszeitpunkt unabhängig mit der 1‑Jahres-Mortalität assoziiert, nicht jedoch das Alter oder die Anzahl der Organversagen (Tab. 4).

**Table 4:** 

Variable	Hazard Ratio (Tod) (95 %-KI)	*p* (1-Jahres-Mortalität)
Alter		*NS*
Anzahl Organversagen		*NS*
Dialyse zum Entlasszeitpunkt	1,13 (1,37–6,95)	*0,007*

*NS* nicht signifikant

## Diskussion

Unsere Studie beschreibt die 1‑Jahres-Mortalität und Dialysepflichtigkeit zum Entlasszeitpunkt und nach 18 Monaten nach einem akuten dialysepflichtigen Nierenversagen auf einer Intensivstation von 118 Patienten. Für ARDS [19], Sepsis [12] oder den kardiogenen Schock im Rahmen eines Myokardinfarkts konnte bereits eine erhöhte 1‑Jahres-Mortalität gezeigt werden [1, 5]. Unsere Ergebnisse decken sich mit zurückliegenden Publikationen, welche eine erhöhte Mortalität bei Patienten mit schwerer AKI auch über den Krankenhausaufenthalt hinaus aufzeigten [1, 11, 13, 16]. In unserer Kohorte betrug die Krankenhausmortalität 45,8 % und stieg ein Jahr nach Krankenhausentlassung auf insgesamt 66,1 % an. Die Dialyse zum Entlasszeitpunkt war in der Cox-Regressionsanalyse ein unabhängiger Prädiktor für eine erhöhte 1‑Jahres-Mortalität. Vor allem Patienten, welche im Rahmen einer Sepsis ein dialysepflichtiges Nierenversagen entwickelten, hatten eine höhere Wahrscheinlichkeit, zum Entlasszeitpunkt nicht mehr auf ein Nierenersatzverfahren angewiesen zu sein.

Die Nachsorge von ambulant versorgten Patienten nach Krankenhausentlassung gestaltet sich oft schwierig. Diese Daten sind jedoch essenziell, um Aussagen über eine potenzielle Erholung der Nierenfunktion im Verlauf treffen zu können. Unsere Studie gewährleistet mithilfe von Fragebögen und einer telefonischen Kontaktaufnahme ein Follow-up von 100 %. Hickson et al. konnten in einer ambulanten Nachsorge zeigen, dass sich die Nierenfunktion nach dialysepflichtigem AKI in ca. 21 % der Patienten innerhalb der ersten 6 Monate wieder erholt [8]. In unserer Studie war bei 21,7 % der dialysepflichtigen Patienten zum Entlasszeitpunkt im Verlauf über 18 Monate kein Nierenersatzverfahren mehr notwendig.

Die intrahospitale Mortalität in unserer Kohorte betrug 45,8 %. In einer retrospektiven Beobachtungsstudie konnten Korkeila et al. vergleichbare Mortalitätsraten aufzeigen. Hier betrug die Mortalität von 62 Patienten mit dialysepflichtigem Nierenversagen bei Krankenhausentlassung 45 % und 6 Monate nach Entlassung 55 % bzw. 65 % nach 5 Jahren. Studien von Bagshaw et al. oder Uchino et al. berichteten mit 60 % bzw. 63,8 % eine deutlich höhere intrahospitale Mortalität [1, 18].

Mit 35,9 % fiel die Dialyseabhängigkeit der Überlebenden zum Zeitpunkt der Entlassung etwas höher aus als in einigen Studien (10–31 %; [1, 4, 18]). Zu berücksichtigen ist der deutlich höhere Anteil von Patienten mit vorbestehender chronischer Niereninsuffizienz in unserer Kohorte (51,6 % vs. 18,8–36,7 % in den Vergleichsstudien [1, 4, 18]). Ein weiterer Unterschied zu den bisherigen Studien ist ein rein internistisches Patientenkollektiv. In den Vergleichsstudien waren auch postchirurgische Patienten mit einem Anteil bis zu 25 % eingeschlossen [16].

Stads et al. [16] konnten eine Korrelation des Grads der Nierenfunktionsstörung zum Zeitpunkt der Krankenhausentlassung mit sowohl der Gesamtmortalität als auch dem Erhalt der Nierenfunktion im Verlauf bei Patienten mit AKI‑D auf einer Intensivstation aufzeigen. Eine eGFR <30 ml/min/1,73 m^2^ stellte dabei den stärksten Prädiktor zur Mortalität dar. Weiterhin besteht ein etwa 80 %iges Risiko für die Überlebenden nach AKI‑D, eine chronische Niereninsuffizienz Grad 3 zu entwickeln [6]. Das Risiko einer dauerhaften Dialyse wird mit 8–21 % angegeben [2, 7].

## Fazit für die Praxis

- Patienten mit dialysepflichtigem akutem Nierenversagen auf einer Intensivstation weisen sowohl eine hohe intrahospitale als auch eine hohe 1‑Jahres-Mortalität auf.
- Eine Dialysefreiheit zum Entlasszeitpunkt stellt einen prognostisch günstigen Faktor dar.
- Eine engmaschige Überwachung der Nierenfunktion bei Patienten mit wiederhergestellter Nierenfunktion ist essenziell.
